# Effects of Additive Manufacturing Techniques for Cobalt-Chromium Alloys on Opposing Enamel Wear

**DOI:** 10.1055/s-0045-1804887

**Published:** 2025-05-01

**Authors:** Pattrapond Eopsirisuk, Wacharasak Tumrasvin

**Affiliations:** 1 Department of Prosthodontics, Faculty of Dentistry, Chulalongkorn University, Bangkok, Thailand

**Keywords:** casting technique, cobalt-chromium alloys, fixed indirect restoration, selective laser melting, tooth wear

## Abstract

**Objectives:**

This study aimed to examine the wear on opposing enamel caused by additive manufacturing techniques for cobalt-chromium (Co-Cr) alloys. Selective laser melting (SLM) techniques were compared with conventional methods. Cast nickel-chromium (Ni-Cr) alloys were also included for comparison.

**Materials and Methods:**

Four groups of dental alloys were examined ( *n* = 10/group): as-built SLM Co-Cr (CS), heat-treated SLM Co-Cr (CS-H), cast Co-Cr (CC), and cast Ni-Cr (NC) alloys. Surface roughness and hardness of these alloys were initially assessed. Wear test was conducted against human enamel cusps using a chewing simulator (49-N load, 1.6-Hz frequency). Volumetric and vertical enamel wear were measured at 60,000, 120,000, and 240,000 chewing cycles using an intraoral scanner combined with open-source 3D software.

**Statistical Analysis:**

Enamel wear was analyzed using a generalized estimating equation (α = 0.05).

**Results:**

Alloy hardness varied among the groups. NC exhibited the lowest hardness, followed by CS, CC, and CS-H. Throughout the entire test, no significant differences in enamel wear were observed among CS, CS-H, and CC. However, NC caused lower enamel wear than the other groups, with a more pronounced difference observed after 120,000 chewing cycles.

**Conclusion:**

SLM is a promising alternative for manufacturing Co-Cr alloys used in fixed dental prostheses, as it exhibited comparable enamel wear to conventional casting. Moreover, optimized heat treatment enhanced the hardness of SLM-fabricated alloys without increasing enamel wear. However, it is noteworthy that Co-Cr alloys fabricated by any techniques resulted in higher enamel wear than Ni-Cr alloys.

## Introduction

Occlusal tooth wear naturally increases with age due to mastication and tooth contact, with enamel wearing at a rate of 20 to 40 μm per year. 1 This rate can be accelerated by various factors and becomes faster as it reaches the dentin. 2 3 Inappropriate selection of restorative materials can exacerbate tooth wear by increasing the wear rate on opposing teeth, particularly at enamel contact areas. 3 4 5 6 Dental restorations, especially those used on occlusal surfaces, should be wear resistant and compatible with natural enamel. 1 5

When selecting materials for dental restorations, numerous options are available. Metal alloys remain the preferred choice for fixed indirect restorations due to their superior strength and retention. 7 Gold-based alloys were previously favored for their minimal wear on opposing teeth. 8 9 However, rising gold prices have caused a shift in preferences toward more economical options, such as base metal alloys. 10 Among these, nickel-chromium (Ni-Cr) alloys were once widely used for manufacturing metal components in fixed dental prostheses, but their use has recently declined in many countries. 10 11 In contrast, cobalt-chromium (Co-Cr) alloys, known for their excellent mechanical properties, corrosion resistance, and durability, have become more prevalent for these indirect restorations. 12

Co-Cr alloys can be manufactured using various methods since digital manufacturing techniques now emerge as viable alternatives to conventional casting. 13 Selective laser melting (SLM), an additive manufacturing technique, is particularly favored for its precise control over product quality by reducing human errors inherent in casting methods. 14 15 SLM-fabricated Co-Cr alloys exhibit superior mechanical properties and more uniform microstructures compared with those produced by conventional casting. 14 15 16 17 18 However, metal restorations manufactured using the SLM process often exhibit higher residual stress from thermal gradients experienced during fabrication. Postprocessing heat treatment is recommended to alleviate this residual stress in SLM-fabricated alloys. 18 19 20

Although SLM is increasingly adopted for fabricating metal restorations and is gradually replacing conventional methods, there are limited studies exploring the wear behaviors of SLM-fabricated alloys. Previous studies indicate that SLM can produce Co-Cr alloys with enhanced wear resistance compared with conventional casting, 21 22 23 24 and heat treatment following the SLM process can influence their wear resistance. 24 While these studies mainly focus on the wear resistance of restorations, there is a lack of research on how Co-Cr alloys manufactured using these techniques affect wear on opposing enamel. Understanding these effects is crucial for making informed decisions and predicting clinical outcomes.

Therefore, the purpose of this study was to examine the effects of SLM-fabricated Co-Cr alloys on opposing enamel wear, involving both as-built and heat-treated forms compared with those produced by conventional casting. Cast Ni-Cr alloys were also included for comparison. The null hypothesis stated that enamel wear would not differ among various alloys across different chewing cycles.

## Materials and Methods

This study was approved by the Human Research Ethics Committee of the Faculty of Dentistry, Chulalongkorn University (HREC-DCU 2023-125). Four groups of dental alloys were examined ( *n* = 10/group): as-built SLM Co-Cr (CS), heat-treated SLM Co-Cr (CS-H), cast Co-Cr (CC), and cast Ni-Cr (NC) alloys. The details of materials used are shown in Table 1 .

**Table 1:** Compositions of materials used in this study

Alloy	Manufacturing	Product name	Manufacturer	Composition (%weight)
Co-Cr	SLM	SLM nonprecious (NP Premier)	Argen Corp, San Diego, CA, United States	Co 61, Cr 25, Mo 6, W 5, Si <1, Fe <1, Mn <1
Co-Cr	Casting	Auriloy N.P. Special	Aurium Research USA, San Diego, CA, United States	Co 59.5, Cr 31.5, Mo 5, Si 2, C <1, Fe <1, Mn <1
Ni-Cr	Casting	Argeloy N.P. Vita	Argen Corp, San Diego, CA, United States	Ni 72, Cr 15, Mo 9, Al 2, Be 1.8, C <1, Si <1, Fe <1

Abbreviations: Al, aluminum; Be, beryllium; C, carbon; Co, cobalt; Cr, chromium; Fe, iron; Mo, molybdenum; Mn, manganese; Ni, nickel; Si, silicon; W, tungsten.

### Preparation of Alloy Specimens

For SLM-fabricated alloys, CS and CS-H specimens were produced using a metal three-dimensional (3D) printer (NCL-M150, Nanjing Chamlion Laser Technology, China) from a rectangular solid computer-aided design (CAD) file (7 × 6 × 4 mm). The process was conducted under a nitrogen gas atmosphere with the following parameters: laser power of 100 W, laser spot size of 100 μm, laser speed of 400 mm/s, hatch distance of 60 μm, layer thickness of 60 μm, and build orientation set at a 45-degree angle.

After the SLM process, CS-H specimens underwent postprocessing heat treatment in a furnace (RQF1400–7-12, Nanjing Chamlion Laser Technology, China). The process involved heating to 1,100°C at a rate of 15°C/min, holding at 1,100°C for 45 minutes, cooling to 500°C over 12 minutes, and then gradually cooling to 40°C over 30 minutes.

For conventionally cast alloys, specimens were fabricated using the lost-wax technique from rectangular solid wax patterns (7 × 6 × 4 mm). CC specimens were cast at 1,480°C using a centrifuge (Fornax T, Bego, Germany), whereas NC specimens were cast at 1,370°C using a centrifuge (Thermo1Dig, JPD Dental Equipment, Greece). All specimens were then allowed to air-cool to room temperature.

All specimens were sequentially polished using silicon carbide abrasive papers (400–1,200 grit) on a polishing machine (Minitech 233, Presi, France). These specimens were mounted on a metallic holder with acrylic resin and cleaned with an ultrasonic cleaner.

Before the wear test, surface roughness and hardness of alloy specimens were assessed. Average surface roughness (Ra) was evaluated using an optical profilometer (InfiniteFocus SL, Alicona, Austria) at 50× magnification on the center of each specimen. Vickers hardness was measured using a microhardness tester (FM-810, Future-Tech, Japan), with a 200-g load applied for 15 seconds. Four hardness measurements were taken for each specimen.

### Preparation of Enamel Specimens

Extracted human permanent premolars were preserved in a 10% formalin solution. The teeth were initially examined under a stereomicroscope (SZ30, Olympus, Japan) to ensure the selection of healthy enamel cusps free from visible cracks, caries, and enamel abnormalities.

Healthy enamel cusps were sectioned from the teeth using a high-speed handpiece equipped with a diamond bur under water coolant. Each cusp was shaped into a cone (diameter = 5 mm, height = 2 mm) by contouring the lower basal part, while preserving the uncut enamel at the cusp tip area. These cusps were then mounted on metallic holders using acrylic resin, with three reference points marked on the resin to facilitate subsequent measurements. Finally, the specimens were cleaned with pumice followed by an ultrasonic cleaner.

Prior to the wear test, all enamel specimens were scanned using an intraoral scanner (TRIOS3, 3Shape, Denmark) and exported as stereolithography (STL) files for baseline 3D images. Calibration of the intraoral scanner was performed before scanning.

### Wear Testing

**Fig. 1:**
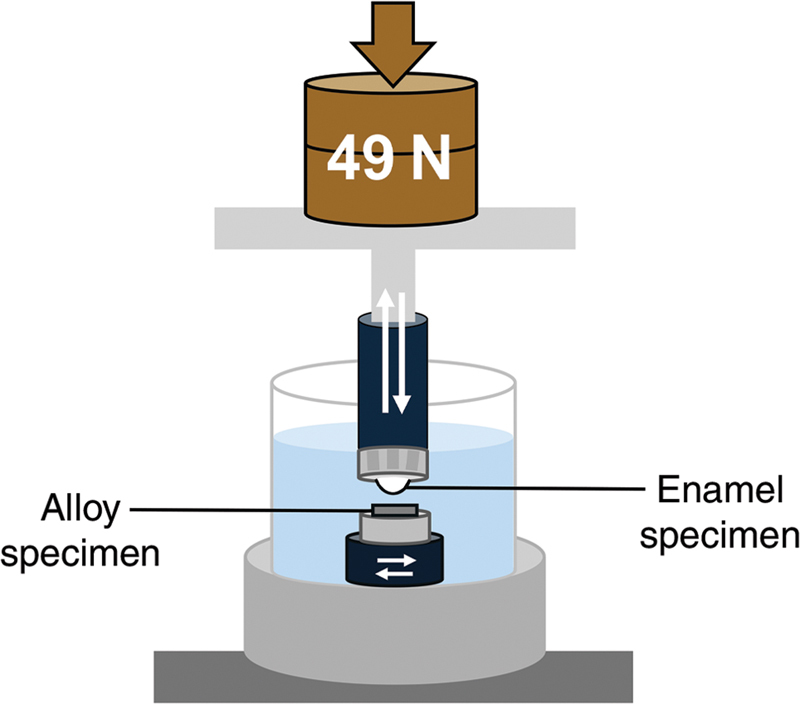
Schematic diagram of chewing simulation.

The wear test of enamel against alloys was conducted using a chewing simulator (CS-4.4, SD Mechatronik, Germany). Enamel specimens were fixed to the upper part, whereas alloy specimens were attached to the lower plate. The chewing simulator operated with vertical movement of the upper part against horizontal movement of the lower plate ( Fig. 1 ). The test was performed in distilled water at room temperature (24°C) with the following parameters: 49-N vertical load, 3-mm vertical stroke, 0.7-mm sliding movement, and 1.6-Hz frequency. A total of 240,000 cycles was completed, simulating 1 year of chewing function, 25 with intermittent pauses occurring at 60,000 and 120,000 chewing cycles. Enamel specimens were scanned at these intervals, with distilled water being replaced in between scans.

### Enamel Wear Measurement

Enamel wear measurement was assessed using open-source 3D software (Blender 3.6.1, The Blender Foundation, the Netherlands). Baseline and subsequent 3D images taken at specific chewing cycles (60,000, 120,000, and 240,000 cycles) were superimposed using the iterative closest point function for best-fit alignment. Volumetric wear and vertical wear were quantified by analyzing the differences in the enamel cusp between baseline and subsequent images.

Remeasurement was performed 2 weeks after the initial measurement to ensure reliability of the analyzing process. Five pairs of images from each group were randomly selected and the entire wear measurement process was repeated.

### Worn Enamel Surface

Enamel specimens were inspected for wear scars using a scanning electron microscope (SEM Quanta FEI 250, FEI, United States) to compare areas with wear scars to those without and to characterize the worn enamel surfaces against various alloys. The 3D surface topography of these enamel surfaces was also examined using an optical profilometer (InfiniteFocus SL, Alicona, Austria) at 50× magnification.

### Statistical Analysis

The data were analyzed using STATA 18.0 (StataCorp, Texas, United States). Normality was assessed using the Shapiro–Wilk test. Baseline surface roughness and hardness of alloys were evaluated using a one-way analysis of variance (ANOVA). Enamel wear was analyzed using a generalized estimating equation. The reliability test was assessed using an intraclass correlation coefficient (ICC). The significance level for all statistical analyses was set at *α* = 0.05.

## Results

Before the wear test, the Ra values of the alloys did not differ among all groups ( *p* = 0.849). However, there was a significant difference in their Vickers hardness ( *p* < 0.001). NC exhibited the lowest hardness, followed by CS, CC, and CS-H ( Table 2 ).

**Table 2:** Surface roughness and Vickers hardness (mean ± standard deviation) of polished alloy specimens before the wear test

Group ( *n* = 10)	Ra		Vickers hardness	
	Mean ± SD (μm)	*p*	Mean ± SD (HV)	*p*
CS	0.106 ± 0.016	0.849	379 ± 7	<0.001
CS-H	0.105 ± 0.009		473 ± 6	
CC	0.109 ± 0.011		431 ± 17	
NC	0.110 ± 0.016		337 ± 17	

Abbreviations: CC, cast Co-Cr alloys; CS, as-built selective laser melting (SLM) Co-Cr alloys; CS-H, heat-treated SLM Co-Cr alloys; HV, hardness value; *n* , sample size; NC, cast Ni-Cr alloys; Ra, average surface roughness; SD, standard deviation.

### Enamel Wear

The cumulative enamel wear in each simulated chewing cycle is shown in Table 3 . At 60,000 cycles, there were no significant differences in either volumetric or vertical enamel wear among all alloy groups ( *p* > 0.05). By 120,000 cycles, NC caused significantly lower volumetric wear compared with CS ( *p* = 0.026) and CS-H ( *p* = 0.004) as well as lower vertical wear compared with CS-H ( *p* = 0.047). After 240,000 cycles, enamel wear against NC exhibited significantly lower volumetric wear ( *p* < 0.001) and vertical wear ( *p* < 0.05) compared with all other groups. Throughout the entire test, no significant differences in enamel wear were observed among CS, CS-H, and CC in each simulated chewing cycle ( *p* > 0.05).

**Table 3:** Cumulative enamel wear (mean ± standard deviation) in each simulated chewing cycle

Group	Volumetric wear (mm ^3^ )			Vertical wear (μm)		
	60,000	120,000	240,000	60,000	120,000	240,000
CS	0.04 ± 0.01 ^a^	0.07 ± 0.02 ^a^	0.13 ± 0.04 ^a^	130 ± 40 ^a^	160 ± 40 ^ab^	220 ± 50 ^a^
CS-H	0.04 ± 0.03 ^a^	0.08 ± 0.03 ^a^	0.13 ± 0.04 ^a^	130 ± 50 ^a^	170 ± 50 ^a^	230 ± 50 ^a^
CC	0.04 ± 0.02 ^a^	0.06 ± 0.02 ^ab^	0.12 ± 0.04 ^a^	130 ± 50 ^a^	160 ± 50 ^ab^	220 ± 50 ^a^
NC	0.03 ± 0.01 ^a^	0.04 ± 0.01 ^b^	0.08 ± 0.02 ^b^	100 ± 30 ^a^	130 ± 30 ^b^	170 ± 40 ^b^

Abbreviations: CS, as-built selective laser melting (SLM) Co-Cr alloys; CS-H, heat-treated SLM Co-Cr alloys; CC, cast Co-Cr alloys; NC, cast Ni-Cr alloys.

Note: Different superscript letters in the same column indicate statistically significant difference ( *p* < 0.05).

Additionally, the ICC for reliability test was 0.998 for both volumetric wear and vertical wear evaluation.

### Worn Enamel Surface

**Fig. 2:**
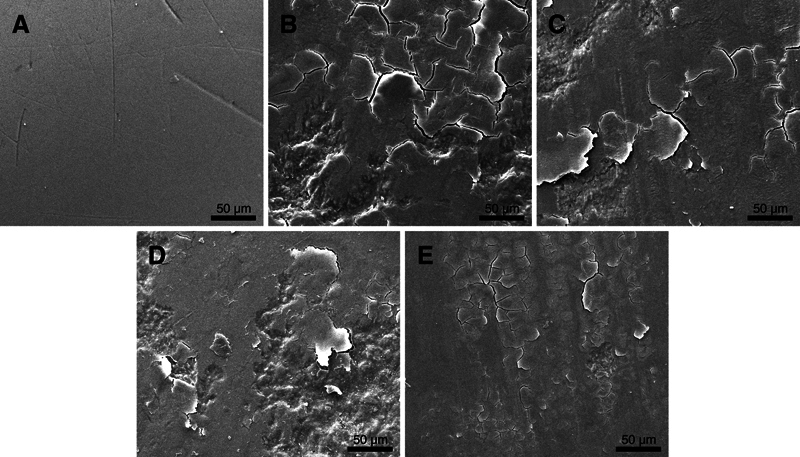
Scanning electron microscope (SEM) micrographs of opposing enamel at 1,000× magnification. ( **A** ) Unworn enamel. ( **B** ) CS, as-built SLM Co-Cr alloys. ( **C** ) CS-H, heat-treated SLM Co-Cr alloys. ( **D** ) CC, cast Co-Cr alloys. ( **E** ) NC, cast Ni-Cr alloys.

**Fig. 3:**
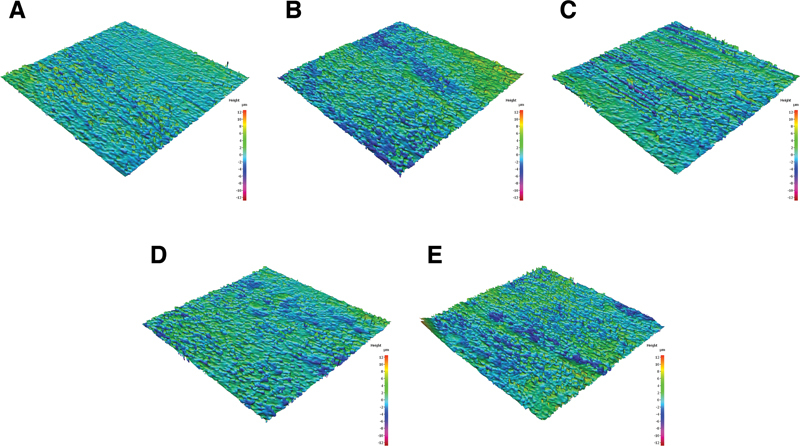
Three-dimensional (3D) surface topography images of opposing enamel at 50× magnification. ( **A** ) Unworn enamel. ( **B** ) CS, as-built SLM Co-Cr alloys. ( **C** ) CS-H, heat-treated SLM Co-Cr alloys. ( **D** ) CC, cast Co-Cr alloys. ( **E** ) NC, cast Ni-Cr alloys.

The unworn enamel exhibited a smooth surface with minor scratches and pits. After the wear test, the enamel surfaces became rougher, showing the presence of cracks and chips. NC caused less surface damage, characterized by furrows with fewer cracks and chips. In contrast, CS, CS-H, and CC resulted in more extensive damage with evident cracks, chipping flakes, and spalling of enamel ( Fig. 2 ). The 3D surface topography images further demonstrated an increased surface roughness of worn enamel compared with unworn enamel ( Fig. 3 ).

## Discussion

Based on the results of this study, SLM-fabricated Co-Cr alloys, in both as-built and heat-treated forms, caused wear on opposing enamel comparable to those produced by conventional casting across all simulated chewing cycles. However, significant differences were observed between Co-Cr and Ni-Cr alloys, particularly evident as the number of cycles progressed to 120,000 and 240,000 chewing cycles. Therefore, the null hypothesis was rejected.

Co-Cr alloys exhibited greater wear on opposing enamel compared with Ni-Cr alloys, which can be attributed to their hardness. In this study, Co-Cr alloys fabricated by any techniques demonstrated significantly higher hardness than Ni-Cr alloys. This increased hardness can result in greater enamel loss when in contact with these alloys. Harder materials are more likely to cause increased wear on antagonists. 26 27 As shown in our findings, Ni-Cr alloys had a greater potential for minimizing wear on opposing enamel since their hardness was lower than that of Co-Cr alloys and closely resembled that of natural enamel, which typically has a Vickers hardness value of 274.8 ± 18.1. 2

Despite variations in hardness among Co-Cr alloys, various manufacturing techniques did not result in different opposing enamel wear. This observation underscores the complexity of wear behaviors in restorative materials, which can be influenced by multiple factors. 26 27 28 Although hardness has been considered a key predictor of wear in metals, 27 29 relying solely on it is insufficient for accurately predicting wear outcomes. 24 Other factors, such as mechanical properties, surface roughness, and microstructural characteristics, also play crucial roles in determining wear behaviors. 30 31 32 33 34 These factors collectively influence their wear resistance and impact on antagonists.

Based on existing knowledge, SLM can produce Co-Cr alloys with enhanced wear resistance compared with conventional casting. 21 22 23 24 However, it is crucial to evaluate their impact on opposing enamel since this serves as an important factor in selecting materials for fixed indirect restorations to prevent excessive tooth wear. This study demonstrated that SLM-fabricated Co-Cr alloys and conventionally cast Co-Cr alloys had comparable effects on opposing enamel wear. Therefore, SLM is a promising alternative to conventional casting for manufacturing occlusal metal restorations in fixed dental prostheses. Moreover, optimized heat treatment is suggested to reduce residual stress in SLM-fabricated Co-Cr alloys without increasing wear on opposing enamel.

Although heat treatment did not affect opposing enamel wear, it influenced alloy hardness. Heat treatment significantly improved the hardness of SLM-fabricated Co-Cr alloys, which can be attributed to changes in phase compositions. Co-Cr alloys typically contain a mixture of face-centered cubic (fcc) and hexagonal close-packed (hcp) phases. 20 Optimized heat treatment increases the proportions of the hcp phase, thereby enhancing strength, hardness, and wear resistance. 18 19 However, the distribution of fcc and hcp phases can vary depending on heat treatment conditions, thus leading to variations in the properties of these alloys. 18 19 20

Manufacturing techniques for Co-Cr alloys also affected their hardness, consistent with prior studies. 15 16 17 18 35 In this study, SLM-fabricated Co-Cr alloys without heat treatment exhibited lower hardness than conventionally cast Co-Cr alloys. While this finding aligns with Choi et al, 35 it contrasts with some previous studies. 15 16 17 18 This discrepancy may be due to variations in SLM process parameters, as any adjustments to these parameters can significantly influence alloy properties. 36 37 38

Although all Co-Cr alloy groups exhibited higher enamel wear than Ni-Cr alloys, the present key finding focuses on the fabrication techniques of Co-Cr alloys. Our study implies that additive manufactured metallic prostheses with proper polishing can be a viable alternative to conventional cast prostheses in terms of effects on opposing enamel wear. These findings support the potential of the SLM technique as a future practical method for fabricating fixed metallic restorations.

## Conclusion

In conclusion, although Co-Cr alloys resulted in greater enamel wear than Ni-Cr alloys, regardless of fabrication techniques, the SLM-fabricated Co-Cr alloys caused wear on opposing enamel comparable to those produced by conventional casting. Therefore, SLM can be a promising alternative for manufacturing Co-Cr alloys in fixed dental prostheses. Moreover, optimized heat treatment can enhance the hardness of SLM-fabricated Co-Cr alloys without increasing opposing enamel wear. However, this study was limited by the specific materials and SLM parameters used. Future studies should explore different materials and process parameters.
